# Developing a Cell-Microcarrier Tissue-Engineered Product for Muscle Repair Using a Bioreactor System

**DOI:** 10.1089/ten.tec.2023.0122

**Published:** 2023-12-11

**Authors:** Ana Luísa Cartaxo, Ana Fernandes-Platzgummer, Carlos A.V. Rodrigues, Ana M. Melo, Katja Tecklenburg, Eva Margreiter, Richard M. Day, Cláudia L. da Silva, Joaquim M.S. Cabral

**Affiliations:** ^1^ Department of Bioengineering and Institute for Bioengineering and Biosciences (iBB), Instituto Superior Técnico, Universidade de Lisboa, Lisboa, Portugal.; ^2^ Associate Laboratory, Institute for Health and Bioeconomy (i4HB), Instituto Superior Técnico, Universidade de Lisboa, Lisboa, Portugal.; ^3^ Medalp Sportclinic, Imst, Austria.; ^4^ Innovacell Biotechnologie AG, Innsbruck, Austria.; ^5^ Centre for Precision Healthcare, Division of Medicine, University College London, London, United Kingdom.; On behalf of the EC Horizon 2020 AMELIE consortium (www.amelieproject.eu). Details of the AMELIE consortium are provided in the Acknowledgments section.

**Keywords:** fecal incontinence, skeletal derived muscle cells, TIPS PLGA microcarriers, myotube formation, CD56

## Abstract

Fecal incontinence, although not life-threatening, has a high impact on the economy and patient quality of life. So far, available treatments are based on both surgical and nonsurgical approaches. These can range from changes in diet, to bowel training, or sacral nerve stimulation, but none of which provides a long-term solution. New regenerative medicine-based therapies are emerging, which aim at regenerating the sphincter muscle and restoring continence. Usually, these consist of the administration of a suspension of expanded skeletal-derived muscle cells (SkMDCs) to the damaged site. However, this strategy often results in a reduced cell viability due to the need for cell harvesting from the expansion platform, as well as the non-native use of a cell suspension to deliver the anchorage-dependent cells. In this study, we propose the proof-of-concept for the bioprocessing of a new cell delivery method for the treatment of fecal incontinence, obtained by a scalable two-step process. First, patient-isolated SkMDCs were expanded using planar static culture systems. Second, by using a single-use PBS-MINI Vertical-Wheel^®^ bioreactor, the expanded SkMDCs were combined with biocompatible and biodegradable (i.e., directly implantable) poly(lactic-*co*-glycolic acid) microcarriers prepared by thermally induced phase separation. This process allowed for up to 80% efficiency of SkMDCs to attach to the microcarriers. Importantly, SkMDCs were viable during all the process and maintained their myogenic features (e.g., expression of the CD56 marker) after adhesion and culture on the microcarriers. When SkMDC-containing microcarriers were placed on a culture dish, cells were able to migrate from the microcarriers onto the culture surface and differentiate into multinucleated myotubes, which highlights their potential to regenerate the damaged sphincter muscle after administration into the patient. Overall, this study proposes an innovative method to attach SkMDCs to biodegradable microcarriers, which can provide a new treatment for fecal incontinence.

**Impact statement:**

At present, no life-long treatment for fecal incontinence exists. New regenerative medicine-based therapies can be seen as a new avenue to solve this burdening health situation. Our approach relies on the administration of implantable biodegradable microcarriers that deliver cells capable of regenerating muscle to the damaged sphincter. In this study, we demonstrate the feasibility of using single-use, bioreactor technology for developing a cell-microcarrier combined product. Moreover, we show that the microcarrier-attached cells, due to their differentiation capacity and the presence of myogenic markers, display muscle regenerative potential, and are likely to be able to provide life-lasting repairs to the damaged muscle.

## Introduction

Damaged skeletal muscle can appear as a result of high-energy road traffic accidents, volumetric muscle loss, sports-related injuries, surgical ablation, and childbirth labor.^1,2^ In the latter, this damage often results in the development of fecal and/or urinary incontinence.^1^ Fecal incontinence is defined as the involuntary loss of rectal contents (feces and gas) through the anal canal and the inability to postpone an evacuation until socially convenient.^3^

Although not life-threatening, fecal incontinence has several consequences for both the patient and their relatives, such as secondary medical morbidities, economic impact by direct and indirect expenses, and reduced quality of life.^1,3^ Since this pathology is usually embarrassing for the patients, it is associated with a social stigma and, consequently, it can be very difficult to obtain trustworthy epidemiology data, as patients end up not reporting it.^3^ Nevertheless, estimates suggest a prevalence of 20% among healthy adult women and 8.6% among older men. In addition, it has been shown to be more prevalent among women and older people.^1,3,4^ In fact, there is a strong correlation between childbirth/obstetric injury and the occurrence of fecal incontinence.^1,3^

Treatment of fecal incontinence starts with nonsurgical approaches such as physical therapy, biofeedback training, and dietary changes.^5^ In the presence of structural deformities, surgical methods, such as sphincteroplasty, colostomy, or sacral nerve stimulation, are applied.^3,4^ These surgical options often lead to several side-effects and complications^3^ that ultimately make the therapy unreliable.^1^ In fact, successful treatments almost always rely on the combination of a number of different approaches.^3^ Importantly, less than 50% of the patients^6^ consider the long-term results of these therapies to be satisfactory. Thus, it is clear that new therapeutic approaches are needed to ensure patient satisfaction.

Regenerative medicine and tissue engineering-based strategies use cells and/or biomaterials to either stimulate the restoration of tissue or to create a tissue mimic in the laboratory that can be later implanted into the patient.^5^ When aiming to restore sphincter muscle function, cells with regenerative potential, such as satellite cells, the putative muscle stem cell population, or their progeny, myoblasts,^1,7^ can be injected into the damaged muscle. However, when an autologous approach is envisaged, the reduced number of cells that can be obtained per biopsy can be a limiting factor.

The first evidence that administration of myoblasts can restore muscle function was shown in an *in vivo* study performed by Partridge et al.^8^ Nonetheless, the pioneer cell therapy clinical study for fecal incontinence was performed by Frudinger et al. using autologous myoblasts.^9^ This work was the first to describe the technical feasibility, acceptability, and clinical effects of myoblast delivery to the sphincter to treat incontinence,^9^ forever changing the paradigm of fecal incontinence treatment.

Since then, additional clinical trials have been conducted, for instances, by Boyer et al.,^10^ in which it was demonstrated that intrasphincteric injections of autologous myoblasts in patients with fecal incontinence showed tolerance, safety, and clinical benefit at 12 months, but not at 6 months. Although positive outcomes have been obtained, these strategies are still far from perfect and there is an urgent need for the development of new, more effective strategies to deliver muscle cells capable of tissue regeneration to the damaged muscle.

This new methodology should avoid both (i) the harsh cell recovery step and (ii) the delivery of these adherent cells as a suspension. Indeed, despite myoblasts being anchorage-dependent cells, the current therapeutic strategies usually deliver these cells as cell suspensions. Therefore, in this context, one alternative is the use of biocompatible and biodegradable microcarriers, which may be implanted into patients. Thermally induced phase separation (TIPS) microcarriers that consist of highly porous microspheres have been previously shown to support the attachment of myoblasts and smooth muscle cells.^11,12^ Scalable inoculation and culture of adherent cells on microcarriers can be performed using different types of bioreactors, such as the recently introduced PBS-MINI Vertical-Wheel^®^ bioreactors (VWBRs).

VWBRs are single-use, scalable vessels that provide gentle culture mixing with more homogeneous shear stress distribution than traditional stirred reactors such as stirred tanks, while being also able to comply with Good Manufacturing Practice (GMP) standards. Being single-use systems, these avoid the need for intensive cleaning/sterilization cycles and possible batch-to-batch contamination.^13^ Moreover, these scalable systems are already proven to be adequate for microcarrier-based culture by ensuring their suspension at low speeds. In fact, this platform has been applied for the culture of several cell types, such as human mesenchymal stromal cells (MSC)^14,15^ and induced pluripotent stem cells.^16,17^

Another fundamental factor to consider when developing a clinical product is the avoidance of animal-origin products to minimize immune reactions and transmission of adventitious agents, as well as the rejection of the administrated product. Moreover, the use of animal-free culture materials increases the likelihood of product approval by the regulatory agencies. Mammalian cells, including skeletal muscle cells, are routinely cultivated using culture medium containing fetal bovine serum (FBS).^18^ Ethical concerns and high risk of contamination with animal products, among others, have been associated with FBS,^19^ reducing its acceptance in a clinical scenario and prompting the search for new growth supplements. Human platelet lysate (hPL) has been proposed as an alternative medium supplement to FBS.

In this study, we propose a xeno(geneic)-free method employing the VWBR to produce poly(lactic-*co*-glycolic acid) (PLGA) TIPS microcarriers cellularized with skeletal-derived muscle cells (SkMDCs) suitable for investigation as a potential innovative therapy for fecal incontinence. The use of implantable and biodegradable microcarriers provides a method of delivering anchorage-dependent cells in a more natural, anchored state. Moreover, it can be implemented in an autologous setting, reducing immune rejection of implanted cells.

The xeno-free methodology combined with the use of GMP-compliant single-use PBS MINI VWBR allows the sterile collection of cells before patient administration in a clinical setting, which increases the likelihood of approval of the final product by regulatory bodies. To demonstrate proof-of-concept for the new process with autologous cells, SkMDCs from three different donors were tested. The methodology developed enabled attachment of SkMDCs onto TIPS PLGA microcarriers with up to 80% efficiency. The adhered cells were found to be viable, maintaining both their myogenic features and their differentiation ability, highlighting their potential to regenerate muscle tissue upon administration and cell engraftment into the host tissue.

## Methods

### SkMDC expansion under static conditions

SkMDCs were isolated (following a previously established methodology, described elsewhere^20^) from muscle tissue biopsy samples from healthy donors, upon signed informed consent and following the principles of the Declaration of Helsinki and of the International Conference on Harmonization—Good Clinical Practice. Before experiments, SkMDCs were thawed and plated on tissue culture flasks (T-flasks; Falcon) at a cell density of 17,000 cell/cm^2^. SkMDCs were cultured with Ham's F10 (Gibco), supplemented with 10% v/v of γ-irradiated FBS (Gibco), 2.4 ng/mL of basic fibroblast growth factor (bFGF; PeproTech), and antibiotic-antimycotic (A/A) (1 × ; Gibco). Cells were maintained at 37°C and 5% CO_2_ in a humidified atmosphere and the culture medium was changed every 2–3 days. At 70–80% cell confluence, SkMDCs were detached from the T-flasks using Trypsin 0.05% (v/v; Gibco) for 4 min at 37°C. Cell number and viability were determined using the NucleoCounter^®^ NC-200 (ChemoMetec).

After thawing, SkMDCs were expanded for at least one cell passage and until reaching the desired cell numbers. SkMDCs from three different donors (characteristics detailed in Table 1) were used in passages 1 (donor 1), 3 (donor 2), and 5 (donor 3).

**Table 1.** Characteristics of Skeletal-Derived Muscle Cell Donors Used in the Developed Work

Donor ID	Donor age, years	Donor gender	Collection site	Cell passage
1	49	Male	Hamstring (*M. Gracilis*, *M. Semitendinosus*)	1
2	18	Male		3
3	48	Female		5

### Preparation of SkMDC-TIPS PLGA microcarrier combination in the VWBR

TIPS PLGA microcarriers were wetted to increase the hydrophilicity and allow cell adhesion. A wetting solution consisting of 77% (v/v) Hanks' Balanced Salt solution (Gibco), 9% (v/v) hPL (nLiven^®^; Sexton Biotechnology), and 14% (v/v) of 70% ethanol solution (*Fábrica do álcool*) was prepared. The wetting solution was added to the vial containing 100 mg of TIPS microcarriers and incubated at 37°C for 72 h on a roller mixer. After this, microcarriers were washed twice with phosphate-buffered saline (PBS) and kept in PBS at 4°C until further use within 72 h.

**FIG. 1.**
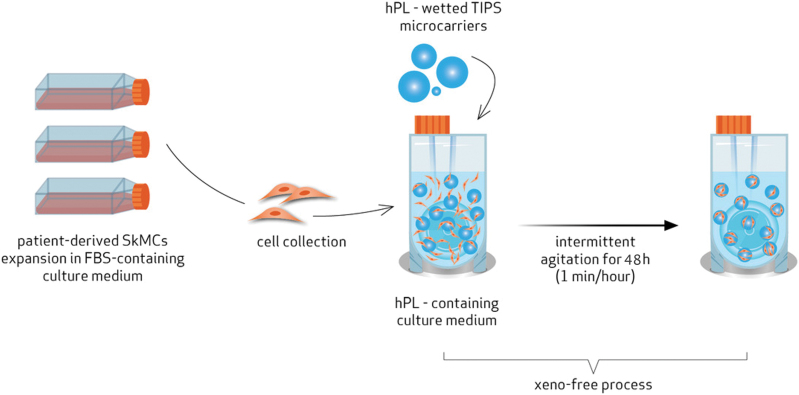
Workflow of the proposed two-step production of a combined product consisting of expanded SkMDCs attached to TIPS PLGA microcarriers. First, SkMDCs are expanded in a planar system using FBS-based culture medium. These cells are then combined with biocompatible and biodegradable TIPS PLGA microcarriers in a VWBR. The combined xeno-free product is obtained by culture of VWBR in an intermittent agitation scheme, using hPL-based culture medium for 48 h. FBS, fetal bovine serum; hPL, human platelet lysate; PLGA, poly(lactic-*co*-glycolic acid); SkMDC, skeletal-derived muscle cells; TIPS, thermally induced phase separation; VWBR, Vertical-Wheel^®^ bioreactor. Color images are available online.

First, we conducted several comparative experiments for the different conditions (culture medium, culture time, agitation regimen, etc.) to assess their impact on cell viability and adhesion efficiency to the TIPS PLGA microcarriers. Then, 20 × 10^6^ SkMDCs and the wetted TIPS PLGA microcarriers were inoculated in a PBS 0.1 VWBR (PBS Biotech, Inc.) containing 60 mL of xeno-free culture medium consisting of Ham's F10 supplemented with 20% v/v hPL, 2.4 ng/mL of bFGF, and 1% (v/v) A/A (1 × ). The VWBR was placed in an incubator at 37°C and 5% CO_2_ with a humidified atmosphere. The rotation speed of the VWBR was set at 40 rpm, with an intermittent profile of 1 min agitation per hour (Fig. 1).

## Experiment

### Experimental design

#### Strategy

The presented method was used to produce a combined product, including SkMDCs and TIPS PLGA microcarriers, to be used as an investigational product for the treatment of fecal incontinence. This method was applied for SkMDCs obtained from three different human donors.

#### Characterization of SkMDC-TIPS PLGA microcarrier combination

**FIG. 2.**
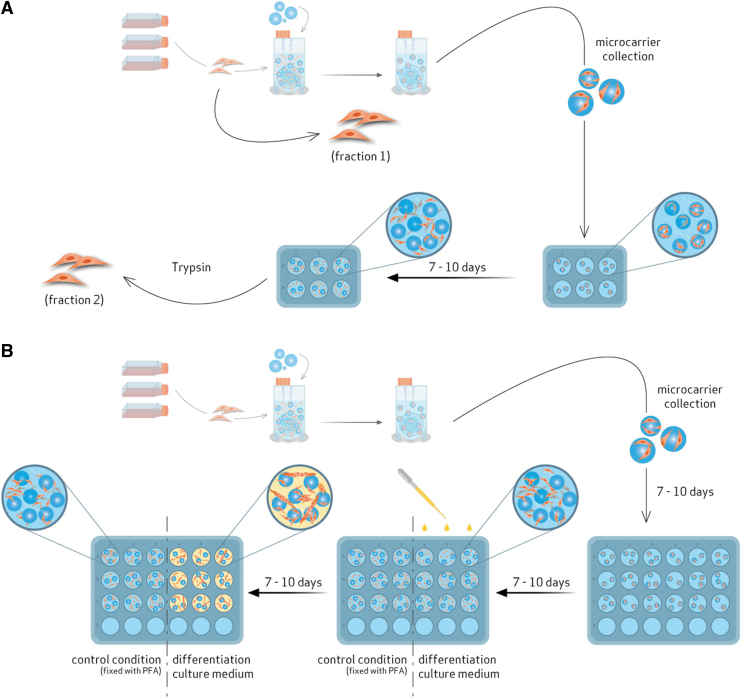
Characterization of SkMDC-TIPS PLGA microcarrier combination: **(A)** Flow cytometry; **(B)** SkMDC differentiation potential. In **(A)**, cell-microcarrier combined product was collected from the VWBR and distributed on a well plate. Then, cells were allowed to migrate to the plate, for 7–10 days. Finally, these migrated cells were collected (fraction 2) and compared with the cells *right* before VWBR inoculation (fraction 1). In **(B)**, cell-microcarrier combined product was collected from the VWBR and distributed on a plate. Then, cells were allowed to migrate to the plate for 7–10 days. After this step, the plate was divided into two different conditions: part is fixed with FA (control condition) and in the other, the culture medium is changed to differentiation culture medium. After 7–10 days, the formation of multinucleated myotubes is evaluated, in the last condition, and compared with the control condition. FA, formaldehyde. Color images are available online.

SkMDC-TIPS PLGA microcarrier combination was characterized by different strategies and techniques (Fig. 2). The quantity of attached and nonattached cells (remaining in suspension), as well as the levels of lactate and glucose present in the culture supernatant, were assessed 24 and 48 h post-VWBR inoculation. In addition, evaluation of cell viability and distribution on the microcarriers was performed by staining the cells with 4′,6-diamidino-2-phenylindole (DAPI, 1.5 μg/mL in PBS; Sigma) and calcein AM (BD Biosciences), at the same time points. At the end of the 48-h period, the whole cell-microcarrier suspension volume was recovered from the VWBR to evaluate different features of the combined product (Fig. 2A, B).

#### Concentration of cells in suspension and attached to the microcarriers

The concentration of cells attached to the microcarriers and cells in the suspension was evaluated at 24 and 48 h post-VWBR inoculation and was quantified using the NucleoCounter NC-200 (ChemoMetec). For that, a cell-microcarrier combination containing sample was collected from the VWBR and transferred to a microfuge tube. Then, microcarriers were allowed to settle and the quantification of cells remaining in suspension was performed. For that, a NucleoCounter cassette was loaded with this supernatant.

For the quantification of attached cells, the remaining supernatant was discarded. Then, a fresh culture medium was added. Reagent A100 (Chemometec) was added in the same proportion of the culture medium. This mixture was incubated for 15 min at room temperature (RT) with point agitation every 2 min. After this incubation time, Reagent B (Chemometec) was added in the same proportion of culture medium. After microcarrier sedimentation, the NucleoCounter cassette was loaded with supernatant. At each time point, at least three independent samples were taken from the VWBR and quantified.

#### Lactate and glucose concentration over time

A VWBR supernatant sample was collected at 24 and 48 h post-VWBR inoculation and was centrifuged at 350 *g* for 10 min at RT. Metabolite concentration (lactate and glucose) was measured with YSI 2500 Biochemistry Analyzer (YSI). Three replicates were collected from the VWBR at each time point.

#### Cell distribution in the microcarriers

Cell distribution in the microcarriers was assessed by microscopy analyses after 24 and 48 h postinoculation. A sample of cell-microcarrier combination was transferred from the VWBR to a tissue culture plate. For DAPI staining, cells were fixed with 4% (v/v) formaldehyde (FA; Sigma) for 15 min. The microcarriers were washed twice with PBS and incubated with DAPI for 45 min under agitation at RT. For calcein AM staining, a different sample was collected. The supernatant was removed and a diluted solution of calcein AM was added to the microcarriers and incubated for 45 min under agitation at RT. The stained cells were visualized using a Leica DMI3000 B microscope Leica SP5 TCS confocal inverted microscope and Leica Microsystems CMS GmbH, Manheim, Germany.

#### Evaluation of myogenic features by flow cytometry

Myogenic characteristics of SkMDCs were evaluated by the expression of CD56^21^ through flow cytometry at two different time points in the process: (i) after cell expansion (and before VWBR inoculation) and (ii) after replating cellcontaining microcarriers and subsequent cell migration from the microcarriers (Fig. 2A, fractions 1 and 2, respectively).

For fraction (1), a sample of cell suspension was collected after cell expansion using T-flasks and processed for flow cytometry staining (Fig. 2A, fraction 1).

For fraction (2), after 48 h cell in the VWBR, a sample of cellularized microcarriers was collected and placed on a tissue culture well plate with fresh xeno-free culture medium. Cell migration from the microcarriers onto the plate was allowed to occur for 7–10 days and the culture medium was changed each 2–3 days. After this time, cells were harvested with trypsin 0.05% at 37°C for 4 min, centrifuged at 350 *g* and resuspended in PBS, and then processed for flow cytometry staining (Fig. 2A, fraction 2).

For both fractions, (1) and (2), 150,000 cells were placed in 2 different microfuge tubes for flow cytometry assays. One tube was used as a control, unstained sample. The other one was incubated with anti-CD56 FITC-conjugated antibody (Biolegend) for 15 min, at RT, in the dark. Then, cells were washed with PBS, centrifuged at 350 *g*, and resuspended in PBS. Flow cytometric analysis was performed using a FACScalibur flow cytometer (Becton Dickinson) and CellQuest™ software (Becton Dickinson) was used for acquisition. A minimum of 15,000 events were acquired for each sample. Data analysis was performed using FlowJo V10 software, in which the unstained population was used to define the gates for the CD56^−^ cell population. These gates were then applied to the stained sample to locate and quantify the CD56^+^ cell populations.

#### Evaluation of myotube formation by immunocytochemistry

After the 48-h adhesion phase in the VWBR, a sample of cell-containing microcarriers was taken and placed on a tissue culture plate containing fresh xeno-free culture medium. Cell migration from the microcarriers to the plate was allowed to occur for 7–10 days and the culture medium was changed each 2–3 days. At this point, two conditions were established: a control condition where cells were fixed with 4% FA and a second condition, where culture medium was changed to Skeletal Muscle Differentiation Medium (Promocell) and cultured for additional 7–10 days. The culture medium was changed each 2–3 days (Fig. 2B). After this time, cells were fixed with 4% FA.

For both conditions, fixed cells were permeabilized with 0.1% saponin (Sigma) for 1 h at RT, washed twice with PBS, and incubated with a blocking solution of 1% (v/v) bovine serum albumin (Sigma) for 30 min at RT. Cells were then incubated with anti-desmin antibody (dilution 1:100; Santa Cruz), overnight, at 4°C, in the dark. After washing twice with PBS, cells were incubated with the secondary antibody Alexa Fluor 546 (1:200; Invitrogen) for 1 h at RT in the dark, washed twice with PBS, and incubated in the dark for 5 min at RT, with DAPI. As a staining control condition, in a different well, cells were processed equally, except for the incubation with the primary antibody. Images were taken with a Leica DMI3000 B microscope. At least three representative photos were taken at each time point.

#### Statistical analysis

Statistical analysis was performed using GraphPad Prism 9 Software. Results are presented as mean ± standard error of the mean of values obtained from different SkMDC donors (i.e., biological replicates). First, normality tests were performed. Then, two-tailed *t*-tests were applied to evaluate the statistical significance of differences in (i) percentage of adhered cells to the TIPS PLGA microcarriers, (ii) glucose concentration, and (iii) lactate concentration, at 24 h versus 48 h for each SkMDC donor. Differences were considered significant at *p*-value <0.05.

### Experimental results

#### SkMDC expansion under 2D conditions

**FIG. 3.**
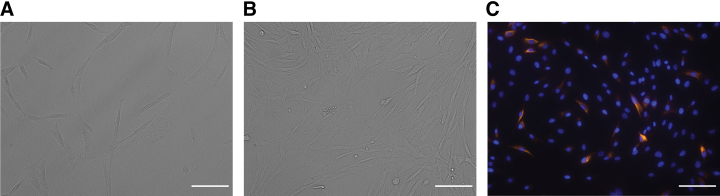
Donor-derived SkMDC expansion in a planar system. **(A)** 3 days postseeding; **(B)** 6 days post seeding; **(C)** desmin and DAPI staining 6 days postseeding. Scale bar: 100 μm. DAPI, 4′,6-diamidino-2-phenylindole. Color images are available online.

The new process proposed in this study begins with the expansion of autologous-derived cells in a planar system (T-flasks). This is equivalent to existing processes used for production of cell-based products for clinical investigations conducted by the provider, Innovacell.^22,23^ The cells investigated were derived from three different donors. The SkMDCs showed a typical stretched and elongated morphology (Fig. 3A, B) throughout the culture period. Desmin staining was performed to confirm that the expansion culture conditions did not induce SkMDC differentiation. Desmin has been described to be synthesized only in fusing or multinucleated cells: its protein levels are reduced in myoblasts and increased in myotubes.^24,25^ We observed that very few cells expressed desmin (Fig. 3C), corroborating that the cells tend to maintain their undifferentiated status, while undergoing expansion under 2D conditions.

#### SkMDC adhesion to TIPS PLGA microcarriers

After expansion, SkMDCs were mixed with TIPS PLGA microcarriers and inoculated into the VWBR to promote cell adhesion. A xeno-free approach was developed to comply with best practice for a product intended for clinical translation. Both TIPS PLGA microcarrier wetting and the cell attachment process were performed under FBS-free conditions by switching to hPL and bFGF supplementation. The cell attachment phase was performed with intermittent agitation to promote cell and microcarrier mixing during the agitation phase and cell attachment during the static phase.

**FIG. 4.**
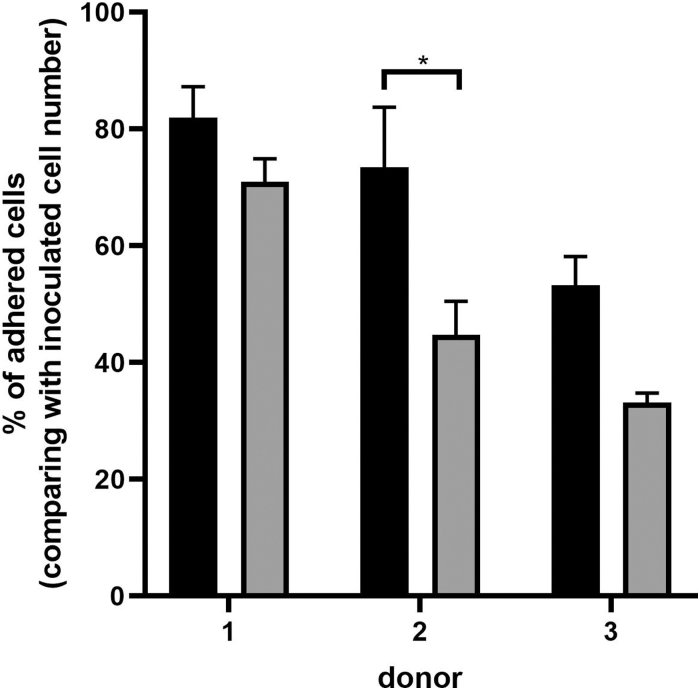
Microcarrier adhered cell concentration over culture time for three different donors. *Black bars* refer to 24-h culture time, and *gray* to 48-h culture time in the VWBR. Results are presented as mean ± SEM (*p < 0.05). SEM, standard error of the mean.

By applying this agitation scheme, considerable cell attachment efficiency was achieved, although with some variability between donors (53–82%, for all donors) (Fig. 4). The highest cell attachment efficiency was obtained after 24 h and was in the range of 70–80%, except for donor 3. The cells remaining in suspension after the same period represented ∼5% of the total inoculated cells. After 48 h, the values of adhesion efficiency, as well as the concentration of cells in suspension, maintained or decreased. In addition, we observed that when applying continuous agitation, cell adhesion efficiency was approximately half the values attained with intermittent agitation (data not shown).

Envisaging the clinical application where high cell numbers are required, large-scale experiments were performed. In these studies, we applied the same protocol, using cells from two donors, and used an average of 70 million cells combined with 500 mg of microcarriers. We could observe similar results in terms of cell viability and adhesion efficiencies, demonstrating that the proposed process is scalable (data not shown).

**FIG. 5.**
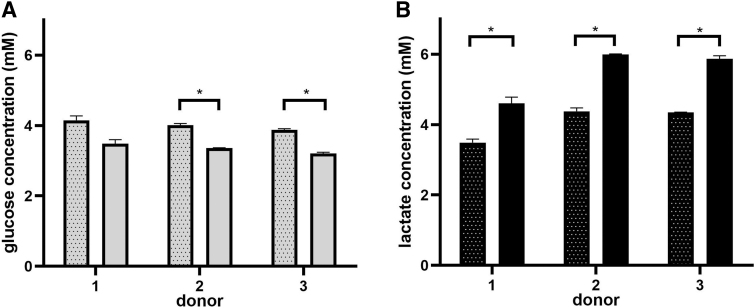
Glucose and lactate concentration over culture time for three different donors. **(A)** Glucose concentration; **(B)** lactate concentration. *Dotted bars* refer to 24 h, while *filled bars* refer to 48-h culture time. Results are presented as mean ± SEM of at least two technical replicates (*p < 0.05).

To evaluate if glucose depletion and/or lactate accumulation was the reason why the attached cells decreased after 48 h versus 24 h, the levels of nutrient/metabolite were measured at both time points. Assuming that the lactate inhibitory concentrations of SkMDCs could be on the same range of MSC (35.4 mM),^26^ it is likely that lactate never reached inhibitory concentrations under the tested experimental conditions (Fig. 5). Glucose concentration was always superior to 3 mM, which does not correspond to a depletion state since some reported culture medium used to cultivate human SkMDCs contain only 2 mM glucose.^27^

#### SkMDC-microcarrier combined product

**FIG. 6.**
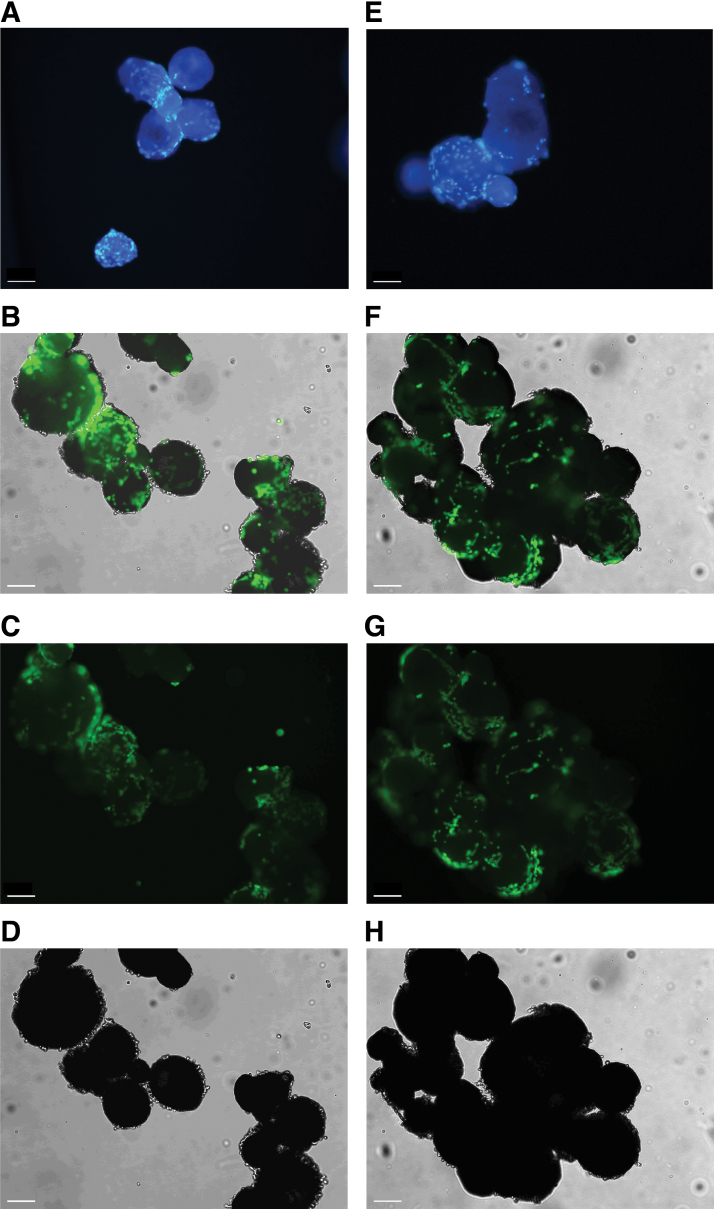
Cell viability and distribution within the microcarriers at 24 and 48 h post-VWBR inoculation. Cell nuclei were stained with DAPI and metabolically active cells were stained with calcein AM. Images were acquired using a fluorescence microscope. **(A–D)** Refers to the 24-h culture time. **(E–H)** Refers to the 48-h culture time. **(A, E)** DAPI staining; **(B, F)** merge of calcein AM and bright field; **(C, G)** calcein AM staining; **(D, H)** bright field. Scale bar: 100 μm. Color images are available online.

To qualitatively evaluate cell viability and distribution on the microcarriers, microscopic analyses were performed. We observed that, for all donors, cell colonization appeared to occur only on the surface of all microcarriers. Cell attachment tended to occur in specific regions of the microcarrier surface (Fig. 6A, E). Attached SkMDCs were viable during the entire evaluated time, as verified by the calcein AM staining (Fig. 6B and F for 24 and 48 h postinoculation, respectively). Some microcarrier aggregation was observed for the three cell donors studied (Fig. 6).

**FIG. 7.**
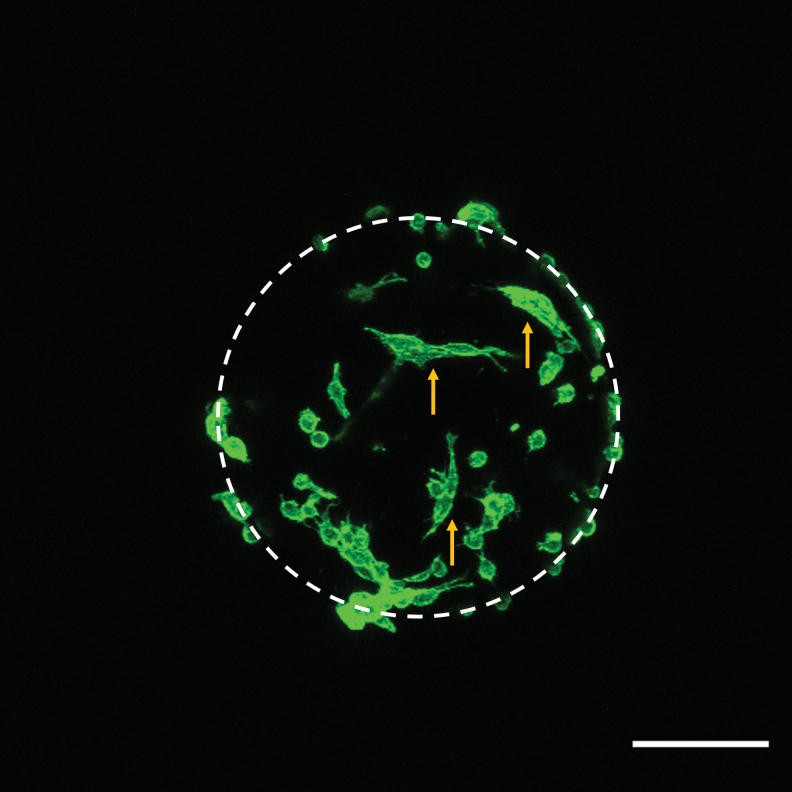
Cell distribution and morphology, while attached to the TIPS PLGA microcarriers. After 24-h adhesion period, cell-microcarrier combined product was fixed with 4% FA, cytoskeleton was stained with phalloidin, and evaluated by confocal microscopy. Scale bar: 100 μm. *Dashed line* indicates the position of microcarriers. *Yellow arrows* point to stretched cells. Color images are available online.

To better characterize cell morphology and distribution throughout the microcarriers, cell-containing microcarriers were also evaluated using confocal microscopy at 24 h post-VWBR inoculation (Fig. 7). We observed that cells were heterogeneously distributed across the microcarriers and some cells exhibited a circular morphology, while others already acquired a stretch-like shape, characteristic of adherent cells.

#### Myogenic regeneration potential of SkMDCs following attachment to TIPS PLGA microcarriers

After the attachment process to the microcarriers, SkMDCs were characterized in terms of their potential to be used in muscle regeneration. To do so, we evaluated cell myogenic characteristics, by assessing the expression of CD56, an important identity attribute that characterizes SkMDCs, which is used to distinguish this cell population from mesenchymal progenitors with adipogenic potential.^28^ In addition, the capacity of attached cells to migrate from the microcarriers onto culture plates (i.e., mimicking cell migration to the damaged site upon administration) and to differentiate into multinucleated myotubes (which is a measure of muscle regeneration potential) was assessed.

**FIG. 8.**
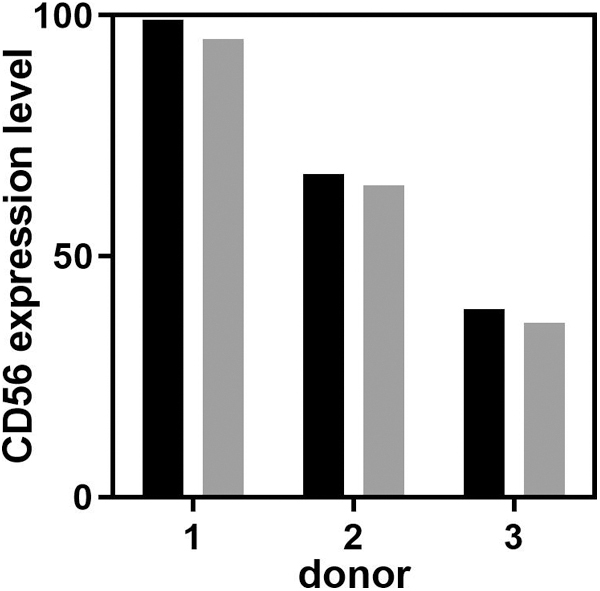
CD56 myogenic marker expression for SkMDCs obtained from the three donors tested. *Black bars* correspond to CD56 levels after 2D expansion, while *gray bars* correspond to CD56 levels after cell migration from the microcarriers.

We evaluated this marker by flow cytometry before VWBR inoculation and after cell migration from the microcarriers (i.e., after replating of cell-containing microcarriers). Results showed that the attachment process did not compromise the presence of the myogenic population essential for muscle regeneration (Fig. 8).

However, we did observe a correlation between cell passage number, cell attachment efficiency, lactate accumulation, and expression of CD56. Donor 3, which was at passage 5, showed the lowest cell attachment efficiency (53%), high levels of lactate accumulation (4.34 and 5.87 mM after 24 and 48 h, respectively), and the lowest values of CD56 expression (36.2% on cells migrated from the microcarriers), under the conditions of our study. Contrarily, donor 1, which was at passage 1, showed the highest cell attachment efficiencies (82%), lower levels of lactate accumulation (3.49 and 4.60 mM after 24 and 48 h, respectively), and highest values of CD56 expression (95% on cells migrated from the microcarriers). Donor 2, which was in passage 3, showed intermediate values for those parameters.

**FIG. 9.**
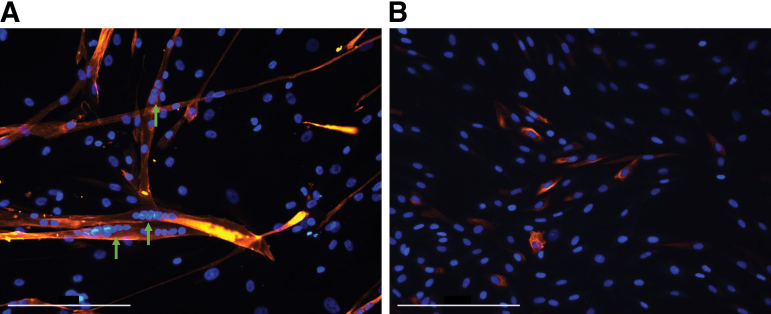
Multinucleated myotube formation assay after SkMDC migration from the microcarriers. Cells were stained with DAPI and desmin. Images were acquired using a fluorescence microscope. **(A)** Cells cultured for 7–10 days in differentiation culture medium; **(B)** control condition. Scale bar: 200 μm. *Green arrows* point to multinucleated myotubes. Color images are available online.

To evaluate the cell-microcarrier combined product performance, SkMDC-TIPS PLGA were plated, and cultured using commercially available SkMDC differentiation culture medium for 7–10 days to allow for cell migration. Similarly, SkMDCs cultured in expansion medium (i.e., nondifferentiation—control condition) were also evaluated. The SkMDCs cultured with differentiation culture medium resulted in the presence of multinucleated myofibers (Fig. 9B); however, the same was not observed in control samples (Fig. 9A), with only mononucleated cells observed similar to those found during the beginning of the culture (Fig. 3C).

## Discussion

Despite the availability of several conventional therapeutic options, there is still an unmet need for a long-term treatment capable of reversing the effects of fecal incontinence.^6^ Most regenerative medicine-based approaches that have been proposed focus on the delivery of a suspension of SkMDCs to the damaged sphincter muscle. Indeed, this strategy is being applied in clinical trials.^29^ Nevertheless, since SkMDCs are intrinsically anchorage dependent, it is likely that approaches using cells in suspension will achieve only suboptimal therapeutic efficacy. In addition, the cell harvesting step for SkMCs from the cell expansion platform can reduce cell viability and impact function.^30^ Also, the injection of cells as a suspension increases the likelihood of cells not remaining at the target site.^31^ Therefore, the development of a new therapy based on injectable biodegradable microspheres carrying functional SkMDCs is highly desirable.

Previous experience from our group has focused on the establishment of bioreactor-based processes toward the development of cell-based therapeutic products. This includes expertise in the use of single-use VWBRs and their application in microcarrier-based cell culture.^15,32^ We also have extensive experience in the establishment of scalable xeno-free processes for the manufacture of human MSC expanded on microcarriers.^19,33^ Furthermore, TIPS PLGA microcarriers have been broadly characterized for various applications, which include myogenesis-related studies.^11,12,34–36^ The work reported herein is the first to combine these areas of expertise to prepare a tissue-engineered combined product with the goal of treating fecal incontinence. This condition still misses a reliable treatment highly due to the inefficient cell delivery typically used in Regenerative Medicine approaches. In fact, these therapeutic approaches rely on delivering a single-cell suspension, associated with low cell viability and cell dispersion from the delivery site.

Moreover, the use of FBS in the several steps of product manufacturing highly hinders those therapies to reach the clinics. With the strategy we present herein, we can overcome all those issues by delivering cells attached to a biodegradable surface, which were prepared under a xeno-free environment. To the best of our knowledge, this is the first report of the culture of human SkMDCs in VWBR using TIPS PLGA microcarriers under xeno(geneic)-free conditions. Our methodology was established by employing (i) biodegradable synthetic polymer microcarriers and (ii) hPL, as an alternative to FBS, to supplement the cell culture medium and the hydration solution for TIPS PLGA microcarriers.

The process proposed herein starts with cell expansion in planar systems to achieve the required cell dose for administration. After one cell passage (following the conditions used in a recent clinical trial^29^), SkMDCs maintained both their characteristic spindle shape and undifferentiated state. In fact, in a clinical setting, one cell passage might be enough to achieve clinically relevant cell numbers depending on the starting number of cells available from the biopsy. In the next step, we conducted cell attachment experiments onto implantable, biodegradable PLGA microcarriers prepared by TIPS. The process of cell attachment was implemented in the VWBR, featuring an innovative low-shear mixing environment, not only because this type of bioreactor enables the culture of shear-sensitive cells but also because it was developed as a single-use system, envisaging compliance with GMP.

After 24 h, the cell attachment efficiency to the microcarriers was ∼70–80%. Due to intrinsic donor-to-donor variability, we observed some variations in the attachment efficiency for the three donors studied. Despite not finding any study in the literature employing the VWBR for SkMDCs, a previous study by our group already used VWBR to expand human MSC combined with plastic microcarriers under xeno-free conditions.^15^ In that work, we obtained adhesion efficiencies of 81% and 49% for MSC derived from adipose tissue and from umbilical cord tissue, respectively, 24 h after inoculation. With the VWBR operation conditions implemented herein, we were able to obtain an attachment efficiency of 70–80% for human SkMDCs on TIPS PLGA microcarriers, which is on the same range of values obtained for adipose tissue-derived MSC.

When testing the feasibility of prolonging the initial phase of the process (i.e., attachment phase of cells to microcarriers), to increase the number of cells attached to the beads, we observed that the cell numbers were either maintained or reduced after 48 h in culture in the VWBR. This suggests that cells did not effectively proliferate during this time period and/or could have detached from the microcarriers. The latter could be potentially explained by either a weak adhesion of cells to the microcarriers, or due to cell dislodgment resulting from cell-microcarrier collisions in the bioreactor vessel. Importantly, SkMDCs were kept viable throughout 24–48 h of stirred culture, with less than 5% of cells found in suspension. Although all observed microcarriers appeared to have cells attached to their surface, their distribution was not homogeneous.

In fact, we observed that cells tended to attach to specific regions of the microcarriers, forming aggregates of cellularized microcarriers. This may arise from the fact that the surface of the microcarriers used is not homogeneous due to their inherent porosity. Alternatively, it could relate to intercellular signaling, resulting in cell migration and clustering. However, other studies with the same type of microcarriers, but different attachment conditions have resulted in homogeneous attachment and distribution of cells,^11,12,37^ which suggests this phenomenon might be unique to the attachment conditions associated with the VWBR or cell type. This trend observed in this study was always observed in the final microcarrier-cell product, regardless of the cell donor. While this aggregation might be responsible for the standard deviation observed in the cell counts, it could also better mimic a tissue construct and avoid microcarrier dispersion from the delivery site.

In this context, it is important to predict how a cell-microcarrier product will behave after administration. For the intended mode of product delivery using a syringe and needle, we have observed that the cell-microcarrier aggregates are weakly bound together and become readily separated into single microcarriers during subsequent processing in readiness for delivery (data not shown). Furthermore, we have observed SkMDC migration from the microcarriers to the surface of a culture plate and that those migrated cells were able to differentiate into multinucleated myotubes. This observation might mimic what would occur upon administration to the damaged tissue target. Based on the type of PLGA and low density of microcarriers created by their high porosity, we expect the microcarriers to degrade over 3–6 months *in vivo* (this is supported by unpublished preclinical safety data).

During this time, the cells are expected to migrate from the surface of the microcarriers and engraft into the host tissue. The proposed mechanism of action for the microcarriers delivering cells to the target site will not be impacted by the longer-term degradation of the microcarriers. From a qualitative standpoint, in the conditions of this study, myotube formation ability was observed for all donor cells, including for donor 3, which had lower CD56 expression compared to other donors. Of notice, Thurner et al. demonstrated that the lack of CD56 expression on the muscle-derived cell's surface correlates with a diminished capacity of myotube formation,^38^ although this trend was not observed in this study.

Overall, this study demonstrates that SkMDCs can be combined with implantable TIPS PLGA microcarriers to produce a xeno-free GMP-compliant product with a high potential for the treatment of fecal incontinence.

The first idea of combining myoblasts/SkMDCs with microcarriers for scalable culture dates back to 1980s,^39^ using beads not intended for implantation. Since then, several works have been published on the topic, describing different microcarrier compositions and shapes (Table 2). Boudreault et al. observed that myoblasts grew on commercially available dextran-based Cytodex^®^ 1 (noncoated) and Cytodex 3 (with a layer of denaturated porcine collagen) microcarriers, but did not grow on polypropylene fabrics or Cellfoam™ macrocarriers, and had an insignificant growth on polyester fabrics.^40^ In another study, Bardouille et al. observed, under static and dynamic conditions, that Cytodex 3 microcarriers were the best to support cell adhesion, proliferation, and differentiation of mouse C2C12 cells, when compared with glass beads and diethylaminoethyl (DEAE) cellulose cylinders.^41^

**Table 2.** Summary of Reports Combining Different Types of Cells with Different Types of Microcarriers

Cell type	Culture conditions	Agitation platform	Culture medium supplementation	Microcarrier type	Ref.
Freshly isolated human myoblasts	Intermittent agitation for the first 4–6 h, followed by the addition of fresh culture medium and then continuous agitation	Spinner flasks	FBS	Cytodex^®^ 1 and 3, polypropylene fabrics, Cellfoam™ macrocarriers and polyester fabrics	^37^
Expanded freshly isolated SkMDCs	Intermittent agitation for 48 h	VWBR	hPL	TIPS	This study
C2C12 murine myoblast cells	Intermittent agitation scheme in the first 3 h, followed by constant stirring. After 24 h, fresh culture medium was added versus static	Superspinner flasks (i.e., featuring an integrated, porous, hydrophobic hollow-fiber membrane)	FCS	Cytodex 3, glass beads and DEAE cellulose cylinders static 2D with 3D dynamic	^38^
Primary murine skeletal muscle cells	Continuous shaking	Cell culture flasks	Neonatal calf serum, FCS and rabbit serum	Gelatin bead microcarriers	^39^
MSC from different sources	Intermittent agitation in the first 6 h, followed by continuous agitation	VWBR	hPL	Xeno-free plastic SoloHill microcarriers	^15^
C2C12 murine myoblast cells	Static versus dynamic conditions	—	FBS	Highly open porous PLGA microspheres	^30^
Human primary skeletal muscle myoblast	Intermittent agitation of 1 min per hour	Ultra-low attachment plates	FBS	TIPS PLGA	^12^
Porcine smooth muscle cells	Intermittent for the first 18 h followed by continuous agitation	Spinner flask	FBS	TIPS PLGA	^40^

DEAE, diethylaminoethyl; FBS, fetal bovine serum; FCS, fetal calf serum; hPL, human platelet lysate; MSC, mesenchymal stromal cells; PLGA, poly(lactic-*co*-glycolic acid); SkMDC, skeletal-derived muscle cell; TIPS, thermally induced phase separation; VWBR, Vertical-Wheel^®^ bioreactor.

By using a different approach, Parmar and Day observed that human SkMDCs attached to TIPS PLGA microcarriers increased in cell number during 5 days of culture following attachment; however, cells did exhibit an elongated morphology similar to this study.^12^ The observed differences with the previous study may be due to the decreased duration of culture (24–48 h) and different mode of agitation used in the bioprocess. Similar morphological features were observed by Kubis et al. at later time points following attachment (16–20 days; on day 9, myoblasts still had a round shape).^42^ Ahmadi et al. also used TIPS PLGA microcarriers combined with smooth muscle cells and demonstrated that cells proliferated, while attached to the surface of the microcarriers for 12 days after spinner inoculation.^43^ Moreover, as observed in our study, smooth muscle cells showed an ability to migrate from the microcarriers to the culture plate.^43^

Other studies have also used PLGA-based microcarriers to cultivate skeletal muscle cells. Kankala et al.^31^ combined PLGA-based microcarriers with mouse myoblasts and reported cell attachment efficiencies ∼60% and ∼40% after 24 h under dynamic and static conditions, respectively. Although their reported adhesion efficiency values were lower than this study, the authors observed cell proliferation from day 1 up to day 16 in both tested conditions, which was not seen throughout 24–48 h of dynamic culture in this study. There are many factors that can affect whether or not cell proliferation on microcarriers is observed during culture. This includes intrinsic cell donor characteristics (donor heterogeneity, background morbidity, and age), the type of bioreactor (configuration, and mode of agitation, exposure to shear forces, and culture medium used), and the assay used to assess proliferation (i.e., direct vs. indirect methods).

Due to the recent surging interest in the production of meat from muscle cells (i.e., cultivated meat), the development of novel microcarrier types and their combination with SkMDCs have become more frequently described in literature (examples of Liu et al. and Andreassen et al.^44,45^). Even though the cells used in these approaches are of bovine origin, the insights attained from their use can be useful and be potentially translated to clinical approaches envisaging muscle repair.

One future step toward the optimization of the manufacturing process for the cell-microcarrier product proposed herein relies on identifying processing parameters that enable SkMDCs to be expanded throughout culture, upon attachment to the microcarriers. This might be achieved by altering the composition of the microcarrier to attain a (functionalized) surface more favorable to cell proliferation and/or optimizing the agitation parameters to ensure that the attached cells do not subsequently detach.

Finally, although hPL is a better alternative to FBS in a clinical translational scenario, it is still far from being an ideal culture supplement, as it has batch-to-batch variability, carries the risk of transmission of human diseases, and raises the possibility of triggering immune responses.^46^ Thus, there is the need to develop alternative culture media, ideally serum-free and chemically defined formulations.

## Conclusion

In this work, we propose a methodology to establish a tissue-engineered product composed of SkMDCs and implantable TIPS PLGA microcarriers as a targeted regenerative approach for treating incontinence. The process described herein was successfully tested with human SkMDCs obtained from different donors, despite some variability in what concerns the efficiency of cell adhesion to the microcarriers. In addition, by a proof-of-concept study, we demonstrated that the process developed herein is scalable, allowing the production of clinically meaningful doses (hundreds of millions of cells *per* dose that are equivalent to previous clinical studies using SkMDCs). Finally, by using single-use vessels and xeno-free culture conditions, we propose the process is able to comply with GMP standards and amenable for use in a clinical scenario. In this context, the technology established herein will be tested in a phase I/II clinical trial under the scope of the AMELIE—*Anchored Muscle cELls for IncontinencE—*consortium (www.amelie-project.eu).
